# Monocular suture needle pose detection using synthetic data augmented convolutional neural network

**DOI:** 10.1007/s11548-025-03467-1

**Published:** 2025-06-24

**Authors:** Yifan Wang, Saul Alexis Heredia Perez, Kanako Harada

**Affiliations:** 1 Graduate School of Engineering, The University of Tokyo, 7-chōme-3-1 Hongō, Bunkyo, Tokyo 113-8656 Japan; 2 Graduate School of Medicine, The University of Tokyo, 7-chōme-3-1 Hongō, Bunkyo, Tokyo 113-0033 Japan

**Keywords:** Needle pose estimation, Robotic-assisted microsurgery, Simulator, Neural network

## Abstract

**Purpose:**

Robotic microsurgery enhances the dexterity and stability of the surgeon to perform precise and delicate surgical procedures at microscopic level. Accurate needle pose estimation is critical for robotic micro-suturing, enabling optimized insertion trajectories and facilitating autonomous control. However, accurately estimating the pose of a needle during manipulation, particularly under monocular vision, remains a challenge. This study proposes a convolutional neural network-based method to estimate the pose of a suture needle from monocular images.

**Methods:**

The 3D pose of the needle is estimated using keypoints information from 2D images. A convolutional neural network was trained to estimate the positions of keypoints on the needle, specifically the tip, middle and end point. A hybrid dataset comprising images from both real-world and synthetic simulated environments was developed to train the model. Subsequently, an algorithm was designed to estimate the 3D positions of these keypoints. The 2D keypoint detection and 3D orientation estimation were evaluated by translation and orientation error metrics, respectively.

**Results:**

Experiments conducted on synthetic data showed that the average translation error of tip point, middle point and end point being 0.107 mm, 0.118 mm and 0.098 mm, and the average orientation angular error was 12.75$^{\circ }$ for normal vector and 15.55$^{\circ }$ for direction vector. When evaluated on real data, the method demonstrated 2D translation errors averaging 0.047 mm, 0.052 mm and 0.049 mm for the respective keypoints, with 93.85% of detected keypoints having errors below 4 pixels.

**Conclusions:**

This study presents a CNN-based method, augmented with synthetic images, to estimate the pose of a suture needle in monocular vision. Experimental results indicate that the method effectively estimates the 2D positions and 3D orientations of the suture needle in synthetic images. The model also shows reasonable performance with real data, highlighting its promise for real-time application in robotic microsurgery.

**Supplementary Information:**

The online version contains supplementary material available at 10.1007/s11548-025-03467-1.

## Introduction

In microsurgery, suturing is one of the most intricate surgical tasks, demanding dexterous and accurate manipulation of the suturing needle. To improve suturing performance in microsurgery, robot-assistant microsurgical systems and suturing skill methods have been widely studied [1], with evidence demonstrating that surgical robots mitigate hand tremors and improve motion precision during suturing. However, robotic suturing at the micro-level remains challenging due to the laborious manipulation of robots. While automation efforts [2, 3] have focused on instrument information, the detection of the suturing needle is out of the research focus. Real-time needle pose estimation is essential for advancing autonomous robotic suturing and improving surgical skill assessment, as accurate pose tracking supports key tasks for needle manipulation, such as grasping [4], regrasping [5] and insertion [6]. Failure to achieve precise needle trajectories can result in tissue damage [7] or complete suturing failure.

The diminutive size of microsurgical needles (e.g., 8-0, 9-0 and 10-0, with chord lengths $\le$ 5 mm and diameters $\le$ 0.04 mm per the USP system [8, 9]) makes sensor-based pose estimation impractical. This limitation has prompted researchers to explore vision-based needle detection and tracking techniques, such as image segmentation [10], ellipse fitting [11] and template matching [12, 13]. Recent advancements in deep learning have further enabled needle detection through methods employing convolutional networks [10, 14, 15]. However, these methodologies often lack the ability to capture precise pose information or depend on stereo-vision systems, which may physically unsuitable for the constrained environments of microsurgery. Furthermore, most surgical microscopes output only monocular video for recording or live streaming, making monocular detection methods necessary for both intraoperative guidance and postoperative evaluation in current clinical setups.

This study proposes a method for estimating the pose of a suturing needle in microsurgery using monocular images. The approach consists of two components: First, a convolutional neural network is employed to detect three keypoints on the needle, namely the tip, middle and end points. Second, the 2D keypoints positions, combined with geometric parameters of the needle, are used to estimate its 3D orientation. The neural network employs an encoder-decoder architecture [16] to generate keypoint confidence maps. This approach is inspired by human pose estimation (HPE) techniques [17], where heatmap-based methods apply 2D Gaussian kernels to ground-truth joint locations [18].

A significant challenge in this approach is the lack of accurately labeled data and spatial information for the suturing needle. This difficulty is handled by generating a synthetic dataset using a VR simulator for robotic microanastomosis. The simulator offers several advantages over manual annotation. (1) It generates ground-truth labeled images hundreds of times faster than manual methods. (2) It ensures highly accurate keypoint labeling, thereby eliminating the errors typically associated with manual annotation. (3) It is capable of labeling occluded keypoints, a task that is unfeasible with manual annotation. (4) It provides accurate ground-truth data for the suturing needle pose, which is crucial for assessing pose estimation accuracy and cannot be achieved through manual methods. Synthetic data augmentation methods, such as GANs [19], CAD modeling [20], and 3D physics simulators [21], are widely used to generate image data for target tasks [22]. From the output confidence maps, the positions of keypoints are determined by locating the local maxima. A custom algorithm then estimates the 3D frame vectors of the needle using 2D keypoint projections, initially providing two potential orientations. By tracking the needle pose over time, the algorithm refines and determines the definitive pose.

Experiments on synthetic images demonstrate the effectiveness of the method in real-time 2D position and 3D orientation estimation, while tests on real images prove its potential for robotic microsurgery applications.

This paper is organized as follows. Section Methods describes the method pipeline of 2D keypoints estimation and 3D orientation estimation. Section Experiments presents the experimental results on synthetic data and real data and related analysis, followed by the discussion Sect. Discussion and conclusion Sect. Conclusion.

## Methods

**Fig. 1:**
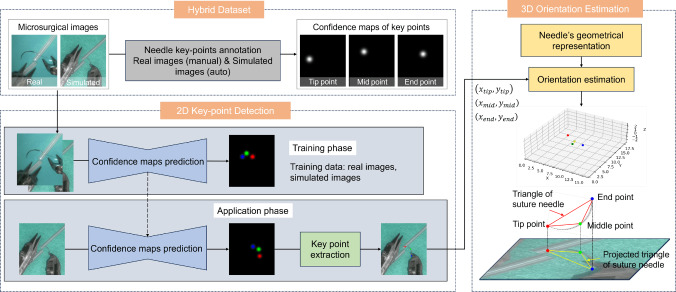
Pipeline of the method to estimate pose of the needle

The pipeline of the proposed method is illustrated in Fig. 1. A hybrid dataset of real and simulated microsurgical images with corresponding confidence maps is used for training a convolutional neural network (CNN) in the 2D keypoint detection phase. The trained CNN then predicts confidence maps of keypoints, from which 2D positions are extracted. These positions, combined with geometric representation of the needle, are used to estimate its 3D orientation.

### Hybrid dataset preparation

**Fig. 2:**
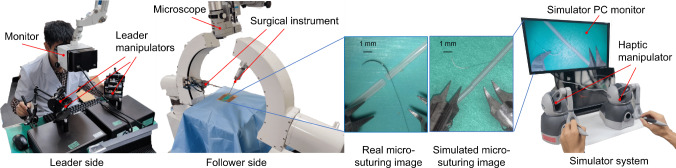
Teleoperated microsurgical robot MM-3 and microscopic images of microvascular anastomosis from real robot and its simulator

Microvascular anastomosis data were gathered using the tele-operated microsurgical robot MM-3 [23] and its simulator to create a hybrid dataset (Fig. 2). The surgical robot in the simulator was controlled via two haptic devices (Phantom Omni, 3D Systems, USA [24]). The virtual anastomosis simulator mimics the physical setup with a green silicone sheet and artificial blood vessels (0.7 mm diameter). This configuration closely replicates the conditions encountered in actual surgical scenarios, ensuring the realism and applicability of the dataset. The simulator was developed using the NVIDIA PhysX engine for dynamic simulation of the needle, deformable vessels and suture. Photorealistic rendering was achieved using the Unity 3D game engine. Accurate CAD models of the tools, needle and vessels were employed to create a high-fidelity virtual scene. The visual properties, including material texture maps and lighting, were manually adjusted to closely replicate the real objects. The virtual camera parameters were matched to the intrinsic parameters of the real camera and positioned accordingly. As a result, the synthetic images closely resembled the real images as depicted in Fig.  2.

The dataset comprises 21 videos, 9 from real surgical robots and 12 from simulations. The real surgical videos, also used for the previous research of needle detection [14], were recorded with a microscope camera, while simulated videos were rendered using the Unity 3D game engine. Each video captures a microsurgical anastomosis trail, with durations ranging from 1 min 37 s to 5 min 14 s, and recorded at a resolution of 470$\times$540 pixels. Frames were extracted at an interval of 25 FPS and then annotated for the tip, middle and end point of the needle to generate confidence maps.

**Fig. 3:**
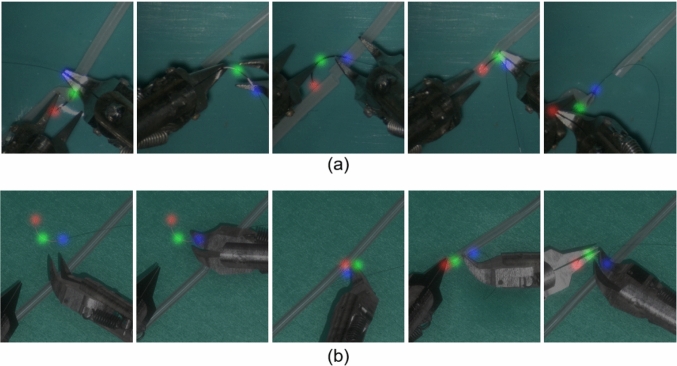
Examples of keypoint annotations for **a** real data and **b** synthetic data. In real data, keypoints cannot be reliably annotated under conditions of severe occlusion. In contrast, synthetic data enables accurate keypoint labeling even under severe occlusion

Manual annotations for real videos were conducted by a trained annotator with prior experience in both microsurgical anastomosis and data annotation. Still, the potential manual error will be introduced during annotation, especially in cases where the needle is partially occluded and keypoints are not fully visible. In such cases, the positions of occluded keypoints were inferred based on temporal continuity across frames and the known geometric structure of the needle unless the keypoints were heavy occluded, which accounted for 4.69%, 3.47%, 3.99% for the tip, middle and end points. In contrast, annotations in simulation videos were automatically derived from ground-truth simulator data during rendering in Unity 3D, enabling accurate keypoint labeling even under full or partial occlusion. Examples of keypoints annotation is illustrated in Fig. 3

Each frame is associated with three confidence maps representing the tip, middle and end points. These confidence maps assign values ranging from 0 to 1 to individual pixels, representing the probability of each pixel being associated with the presence of a keypoint. The probability distribution of the confidence map follows a 2D Gaussian distribution, with the center defined by the coordinates of the keypoints. The 2D Gaussian distribution function can be expressed as

1 $$\begin{aligned} G\left\{ \textbf{x} \right\} =\frac{1}{\sqrt{(2\pi )^{2}\left| \textbf{V} \right| }}\exp \left( -\frac{1}{2}(\textbf{x}-\varvec{\mu } ) \textbf{V}^{-1} (\textbf{x}-\varvec{\mu })^{T} \right) \end{aligned}$$

where $\textbf{x}$ and $\varvec{\mu }$ are vectors representing two variables and their corresponding mean values: $\textbf{x}=\left( x_{1},x_{2} \right)$ and $\varvec{\mu } =\left( \bar{x_{1}},\bar{x_{2}} \right)$, The matrix $\textbf{V}$ represents the variance-covariance matrix:

2 $$\begin{aligned} \textbf{V}=\begin{pmatrix} \sigma _{1}^{2} &  \sigma _{12}^{2}\\ \sigma _{21}^{2}&  \sigma _{2}^{2} \end{pmatrix} \end{aligned}$$

where $\sigma _{1}^{2}$ and $\sigma _{2}^{2}$ denote the variance of $x_{1}$ and $x_{2}$, respectively. And $\sigma _{12}^2$ = $\sigma _{21}^2$ are the covariance between variables $x_{1}$ and $x_{2}$. Modifying the value of $\sigma _{1}^{2}$ and $\sigma _{2}^{2}$ alters the size of Gaussian distribution, thereby influencing the size of focus regions during model training. In constructing the confidence maps, the variances were set to $\sigma _{1}^{2}$=$\sigma _{2}^{2}$= 200 and the covariance to $\sigma _{12}$=$\sigma _{21}$= 0, indicating no linear relationship between the variables. The vector $\mu$ was set as the position of the labeled keypoint. During training, various image augmentation methods were applied, such as: random flip, random crop, color transformation, rotation, distortion and blurring. After the augmentation process, the dataset expanded to a total of 15,000 images. Specifically, 10,000 images were used for training, 2500 for validation and another 2500 for testing.

### 2D keypoint detection

For 2D keypoint detection, a convolutional neural network is trained on the hybrid dataset, which is employed to predict the confidence map of keypoints. Subsequently, the coordinates of keypoints can be extracted and used for 3D orientation estimation.

#### Network architecture

**Fig. 4:**
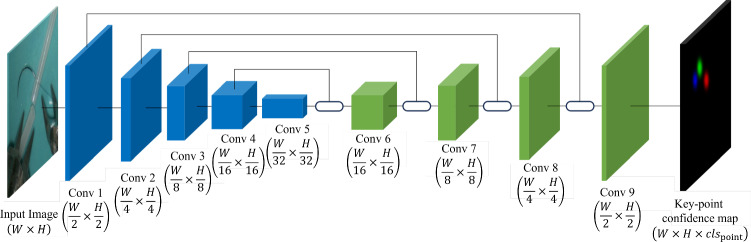
The overview of the network architecture for keypoint confidence map prediction. *Conv* is an abbreviation for convolutional layer, which is a fundamental block in CNN. The number following *Conv* indicates the position of the layer in the network’s sequence

A U-Net-inspired encoder-decoder architecture [16] is employed to predict keypoint confidence maps, as shown in Fig. 4. The full model architecture and implementation details are provided in Appendix A.

#### Network training and evaluation

The mean squared error (MSE) loss [25] is applied for supervision by comparing the predicted confidence map to the ground-truth confidence map pixel-wisely. The learning rate is configured to be 0.0001 during epochs 1 to 30, followed by a reduction to 0.00001 in epochs 31 to 50. The batch size of 4 is used during training, with optimization performed using the Adam optimizer with weight decay of 0.01 [26]. All the architecture was implemented using PyTorch 1.8.0 and CUDA 11.1 based on Python 3.8 and executed in Ubuntu 20.04 with an NVIDIA Quadro P6000 graphics card.

For evaluation of model accuracy, the widely used PCK (percentage of correct keypoints) metrics from pose estimation field [27] is applied. PCK evaluates accuracy by measuring whether the detected keypoint lies within a specified distance threshold from the true keypoint. The metrics of PCK is

3 $$\begin{aligned}  &   \text {PCK} = \frac{1}{N}\sum _{i=1}^{N}\delta _{i}\left( |\hat{p}_{i} - p_{i}| \right) , \end{aligned}$$

4 $$\begin{aligned}  &   \quad \delta _i(x) = {\left\{ \begin{array}{ll} 1 &  \text {if } x < d_{\text {threshold}} \\ 0 &  \text {otherwise} \end{array}\right. } \end{aligned}$$

where *N* is the total number of keypoints, $\delta _{i}$ is a binary indicator function that equals 1 if the Euclidean distance between the detected keypoint $\hat{p}_{i}$ and the ground-truth keypoint $p_{i}$ is less than a specified threshold $d_{\text {threshold}}$ and 0 otherwise. The $d_{\text {threshold}}$ is usually set with respect to the scale of the subject. In this study, $d_{\text {threshold}}$ is set to 5 pixels, approximately twice the needle thickness, ensuring accurate keypoint detections relative to the needle in the images.

The evaluation results of network architecture on test dataset show a performance with PCK reaching up to 96.03% at a speed of 42 FPS, which can meet the requirements of real-time inference.

### 3D orientation estimation

The 3D orientation estimation procedure involves three phases: (1) estimation of candidate needle-frame vectors to identify potential vector pairs, (2) initialization of needle-frame vectors to determine a definitive pair of frame vectors and (3) attainment of specific needle-frame vectors through tracking to maintain consistency across sequential frames.

#### Estimation of potential needle-frame vectors

**Fig. 5:**
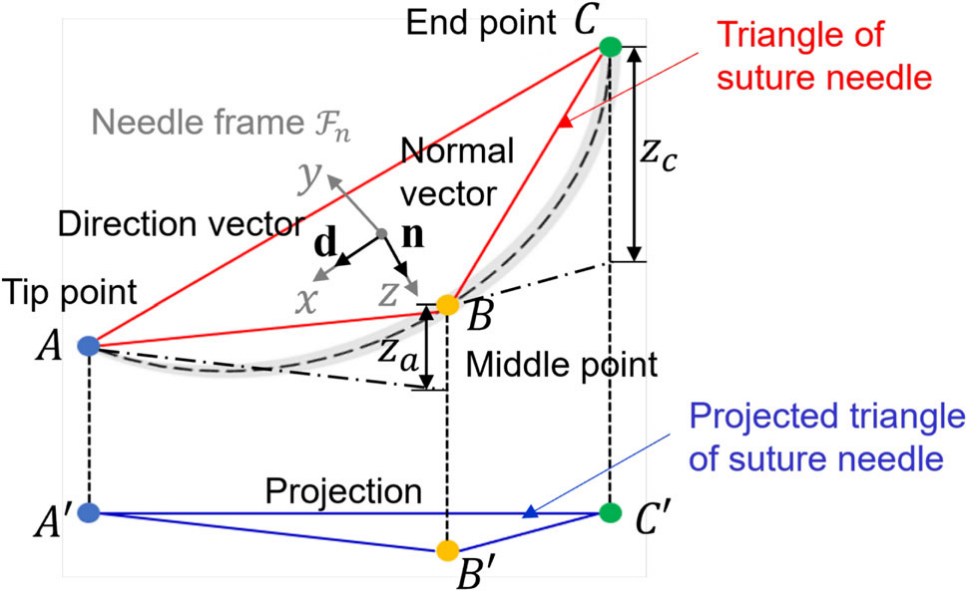
Illustration of keypoints and needle-frame vectors

In the first phase, the algorithm calculates potential needle-frame vectors using keypoints positional information and geometric parameters of the needle. The suture needle, with standardized geometric specifications for medical applications, is geometrically represented as a triangle formed by its tip, middle and end points. Figure 5 illustrates the keypoints and frame vectors of needle. Points *A*, *B* and *C* denote the tip, middle and end point of the needle, respectively, while $A^{\prime }$, $B^{\prime }$ and $C^{\prime }$ represent their projections on image. Given the specifications of the micro-vision camera (focal length: 175 mm, working distance: 875 mm, depth of field: 10 mm), the projection lines $AA^{\prime }$, $BB^{\prime }$, and $CC^{\prime }$ can be assumed to be perpendicular to the image plane $A^{\prime }B^{\prime }C^{\prime }$ (Appendix B). This assumption is justified by the relatively long working distance and narrow depth of field, which contribute to a nearly parallel projection geometry over the observed region. The origin of the needle frame is defined as the centroid of points *A*, *B* and *C*. The needle-frame vectors consist of a normal vector $\textbf{n}$ (z-axis) and a direction vector $\textbf{d}$ (x-axis). The normal vector $\textbf{n}$ is the unit vector of the cross product of $\textbf{CB}$ and $\textbf{BA}$, given by $\textbf{n}=\frac{\textbf{CB}\times \textbf{BA}}{\left| \textbf{CB}\times \textbf{BA} \right| }$. The direction vector $\textbf{d}$ is the unit vector of $\textbf{CA}$, expressed as $\textbf{d}=\frac{\textbf{CA}}{\left| \textbf{CA}\right| }$.

**Fig. 6:**
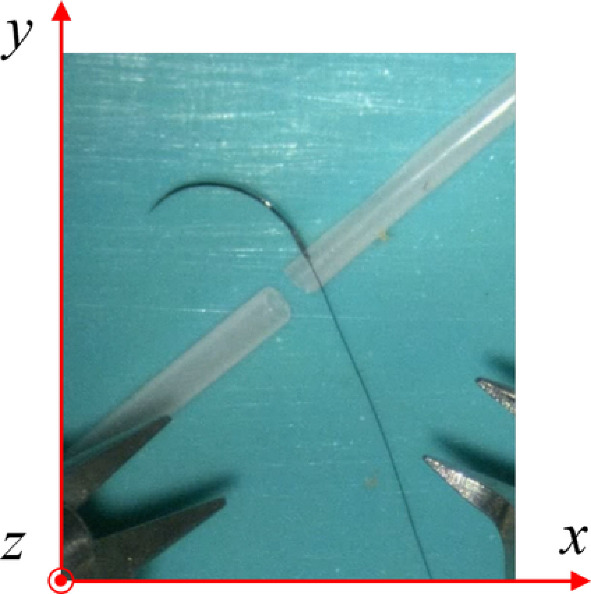
The frame of image used for the needle orientation estimation

Given an input image and its coordinate frame (Fig. 6), the 2D positions of the keypoints: tip point $A^{\prime }$ $(x_{a}, y_{a})$, middle point $B^{\prime }$ $(x_{b}, y_{b})$ and end point $C^{\prime }$ $(x_{c}, y_{c})$ are represented in pixels coordinates. A known-size ruler is incorporated into the image to provide the size ratio $r=\frac{pixel }{real }$, which is consistent along both the x and y dimensions, enabling unit calibration to standardize length measurements between the image frame and real-world dimensions.

Algorithm 1 outlines the computation of the needle-frame vectors. To determine the 3D orientation of the needle, the relative *z* position of points *A*, *B* and *C* must be calculated. Assuming $z_b = 0$ for the middle point, $z_a$ (tip point) and $z_c$ (end point) are obtained using Eq. 5, yielding two symmetrical solutions for $z_a$ and $z_c$. With these values, the vectors $\textbf{CB}$, $\textbf{BA}$ and $\textbf{CA}$ are derived for needle-frame vectors.

5 $$\begin{aligned} \left\{ \begin{matrix} z_{a}EMPTY ^{2}=|AB|^{2}-|A{EMPTY}^{\prime }B{EMPTY}^{\prime }|^{2}\\ z_{c}EMPTY ^{2}=|BC|^{2}-|B{EMPTY}^{\prime }C{EMPTY}^{\prime }|^{2}\\ |z_{a}-z_{c}|^{2}=|AC|^{2}-|A{EMPTY}^{\prime }C{EMPTY}^{\prime }|^{2} \end{matrix}.\right. \end{aligned}$$

**Algorithm 1:**
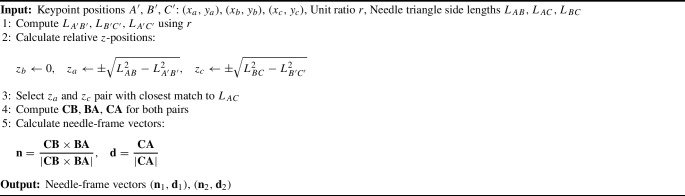
Needle-frame vectors calculation

#### Needle-frame vectors initialization and tracking

**Fig. 7:**
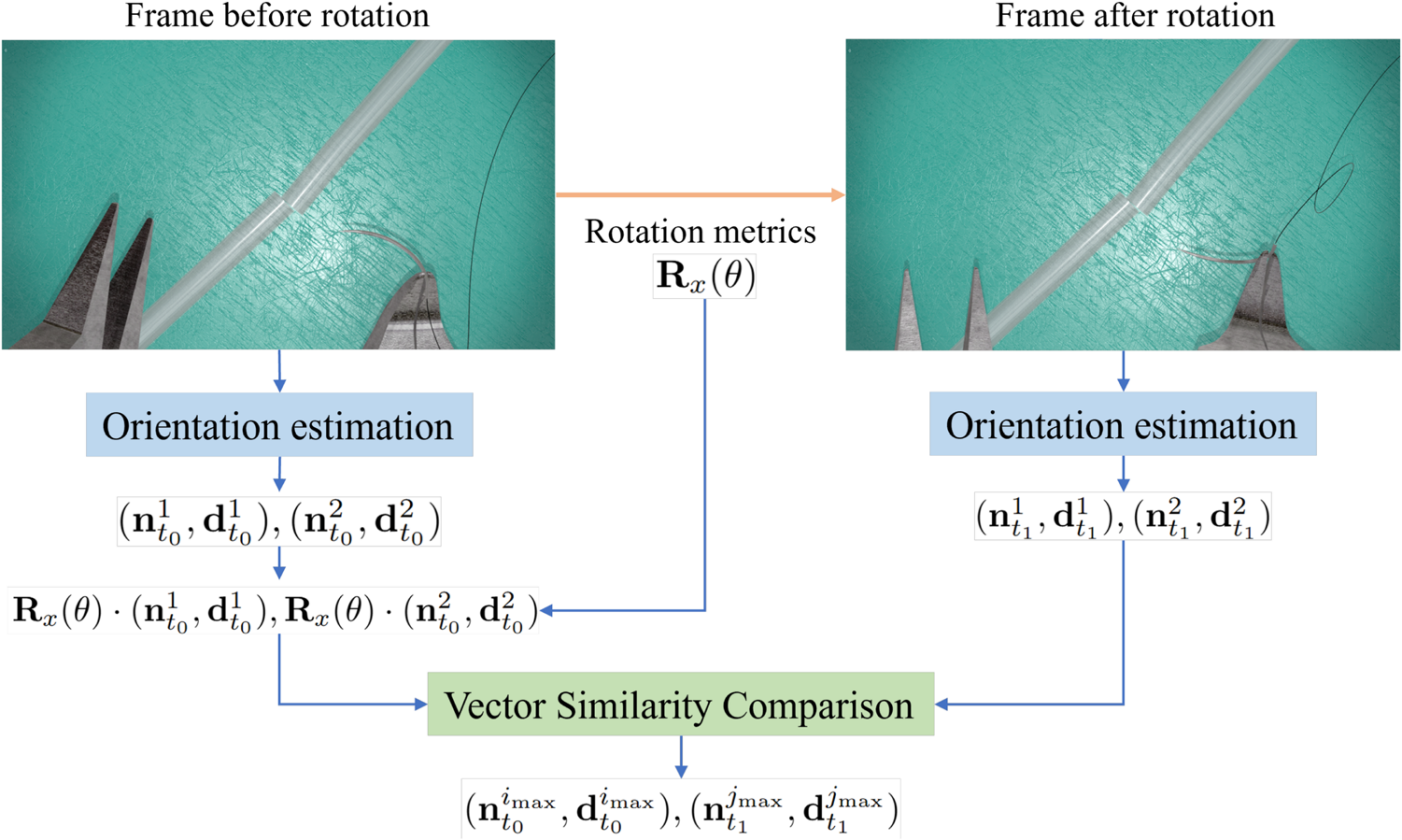
Needle-frame vectors initialization procedure

The estimated needle-frame vector pairs are symmetrical relative to $x-y$ plane of image frame. To identify the correct needle-frame vectors, a tracking-based initialization method was applied, as shown in Fig. 7. First, the forceps attached to the robot end-effector grasp the needle, yielding two possible needle-frame vector pairs at time $t_0$, denoted as $(\textbf{n}_{t_{0}}^{1}, \textbf{d}_{t_{0}}^{1}) , (\textbf{n}_{t_{0}}^{2},\textbf{d}_{t_{0}}^{2})$. Since the needle is rigidly connected to the robot via the forceps, its motion transformation mirrors that of the robot end-effector. If the end-effector rotates around the x-axis of the image frame by an angle $\theta$, the corresponding rotation matrix is

6 $$\begin{aligned} \textbf{R}_x(\theta ) = \begin{bmatrix} 1 &  0 &  0 \\ 0 &  \cos (\theta ) &  -\sin (\theta ) \\ 0 &  \sin (\theta ) &  \cos (\theta ) \end{bmatrix} \end{aligned}$$

needle-frame vector pairs, $(\textbf{n}_{t_{1}}^{1}, \textbf{d}_{t_{1}}^{1})$ and $(\textbf{n}_{t_{1}}^{2}, \textbf{d}_{t_{1}}^{2})$, are obtained. By comparing the similarity of needle-frame vectors at $t_1$ and the rotated vectors of $t_0$: $\textbf{R}_x(\theta )\cdot (\textbf{n}_{t_{0}}^{1}, \textbf{d}_{t_{0}}^{1}), \textbf{R}_x(\theta )\cdot (\textbf{n}_{t_{0}}^{2}, \textbf{d}_{t_{0}}^{2})$, the correct needle-frame vectors at both $t_0$ and $t_1$ can be determined, thereby initializing the needle-frame vectors. The details are shown in Algorithm 2

**Algorithm 2:**
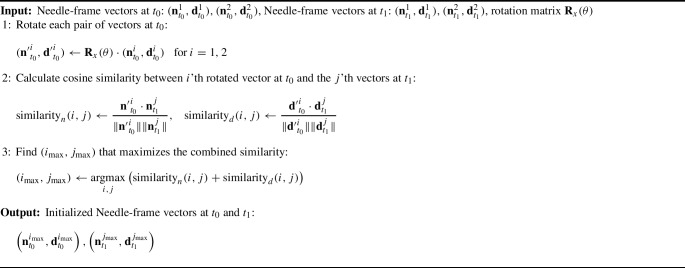
Needle-frame vectors initialization

After needle-frame vectors initialization, subsequent possible needle-frame vector pairs are iteratively compared with the current correct vectors to determine the updated correct needle-frame vectors.

## Experiments

In our experiment, a 10-0 gauge needle with a chord length of 4 mm, a diameter of 100 $\upmu$m and a 3/8 circle curvature was used to suture a blood vessel with a diameter of 0.7 mm. As described in Sect. Estimation of potential needle-frame vectors, the needle was modeled as a triangle with side lengths $L_{AC}=4$ mm and $L_{AB}=L_{BC}=2.4$ mm. For initializing the needle-frame vectors, the rotation matrix was set as $\textbf{R}_{x}(90^{\circ })$, indicating a $90^{\circ }$ rotation of the end-effector around the x-axis in the simulator. This specific rotation was selected based on experimental observations demonstrating that a single-axis rotation about the *x*-axis reliably enabled accurate alignment of the needle-frame vectors without introducing ambiguity. In all conducted trials (6 out of 6), this configuration consistently resulted in successful initialization of the needle-frame vectors. Moreover, such a configuration facilitates improved maneuverability during manual teleoperation by simplifying the rotational motion. After initialization, the keypoints and frame vectors of the needle in subsequent frames were tracked and predicted.

Two experiments were conducted: First, the Euclidean distance error of 2D keypoint positions and angular error of 3D orientations was evaluated using synthetic data, as ground-truth spatial information for real surgical needles is unavailable, making the same evaluation method inapplicable to real data. In the second experiment, error on real data was assessed by measuring the 2D keypoint translation error in pixels and millimeters.

### Experiment on synthetic data

A total of 6 trials were conducted using videos captured from a surgical robotic simulator. Notably, the frames used in these trials were not part of the training dataset. Concurrently, the positions of the needle keypoints were recorded by the simulator, which served as the ground truth for evaluating both keypoint positions and needle-frame vectors.

To evaluate the error of the 2D keypoints positions, an affine transformation was applied to convert the keypoint coordinates from the image frame (pixels) to the simulator frame (millimeters). Translation error was then calculated as the Euclidean distance between the detected and ground-truth keypoints, measured in millimeters.

For 3D orientation evaluation, cosine similarities and angular errors between the estimated and ground-truth needle-frame vectors were computed.

### Experiment on real data

To explore the practicability of the proposed method on real data, 250 real images were collected for testing. Due to the impossibility in acquiring ground-truth spatial information for real surgical needles, direct evaluation of 3D orientation was not feasible. Instead, the performance was assessed by measuring the 2D keypoint translation error in pixels. Utilizing the known pixel-to-millimeter scaling ratio, these pixel-based errors were further converted to estimate the corresponding translation errors in real-world units (millimeters).

**Fig. 8:**
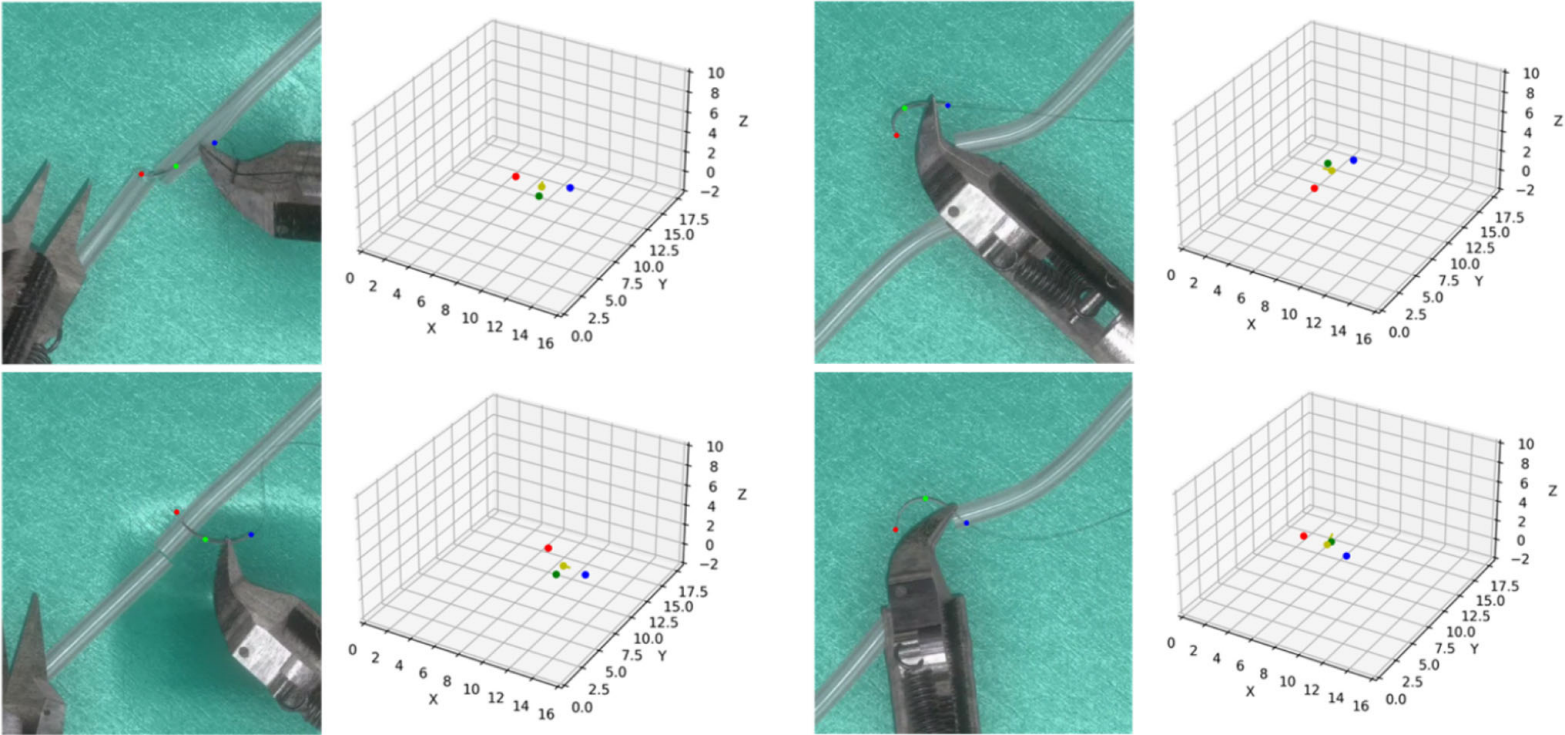
Inference results on synthetic images, the left column are 2D keypoints detections and right column are 3D orientation estimates. Red: tip point; Green: middle point; Blue: end point; Yellow: unit normal vector

### Correlation analysis of keypoints translation error and orientation error

As the 3D orientation estimation relies on the 2D keypoint detections, any errors in keypoint localization will propagate to the 3D orientation estimates. Given that direct measurement of 3D orientation errors on real data is not feasible, these errors can be approximated by analyzing the relationship between keypoint translation errors and orientation estimation accuracy.

**Table 1:** 2D keypoints translation error on synthetic data

	Tip point	Middle point	End point
Translation error (mm)	$0.107 \pm 0.096$	$0.118 \pm 0.092$	$0.098 \pm 0.095$
Detection ratio	94.83%	98.49%	97.38%

The correlation between keypoint translation error (pixel) and orientation angular error was analyzed using 400 synthetic images to investigate the impact of keypoint detection accuracy on orientation estimation. For each image, the average keypoint translation error and angular errors of the normal and direction vectors were calculated. Pearson correlation coefficients were then calculated to quantify the strength of the association between keypoint translation errors and orientation errors.

## Results

### Results on synthetic data

**Fig. 9:**
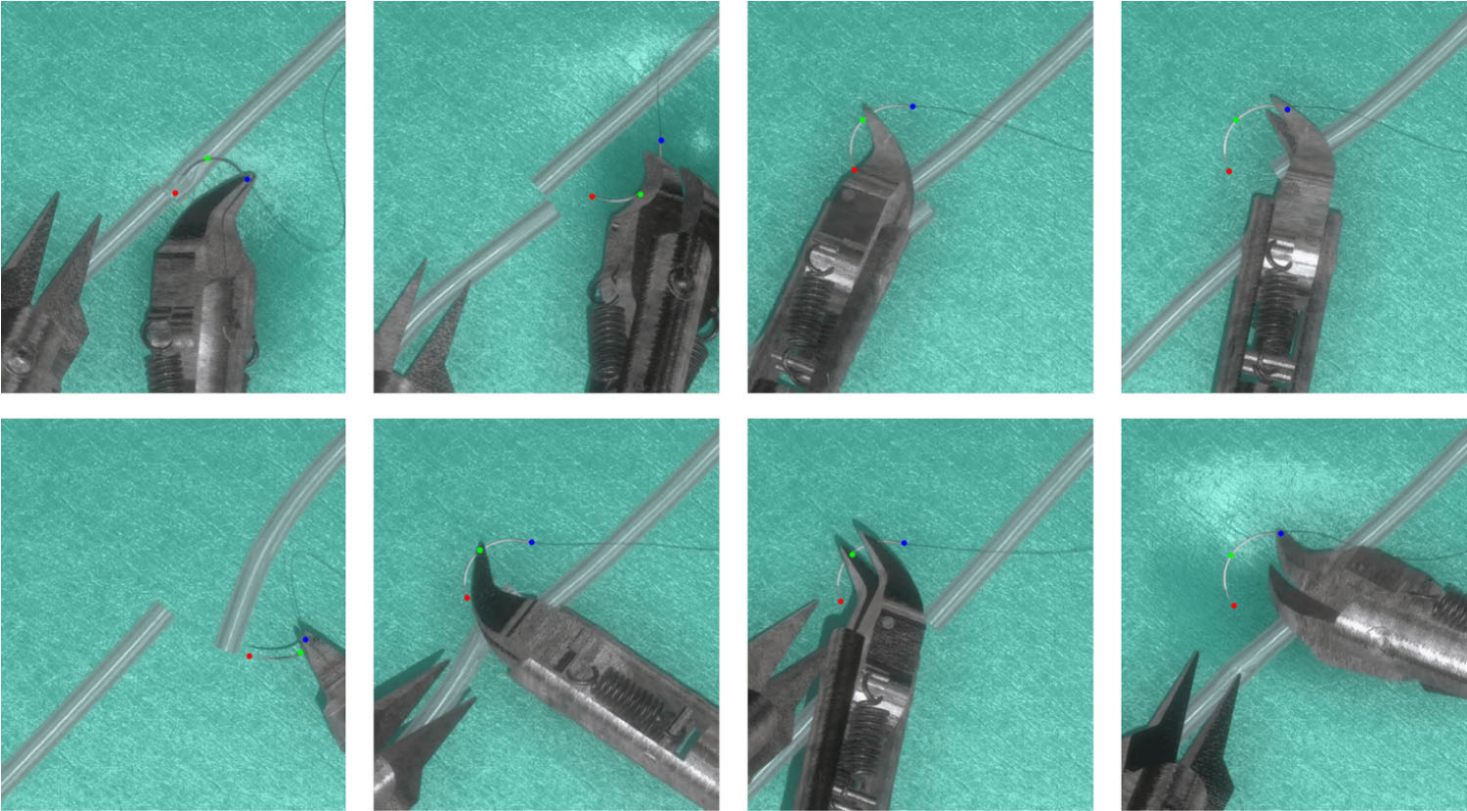
Successful inference results of keypoints under occlusion situations

**Fig. 10:**
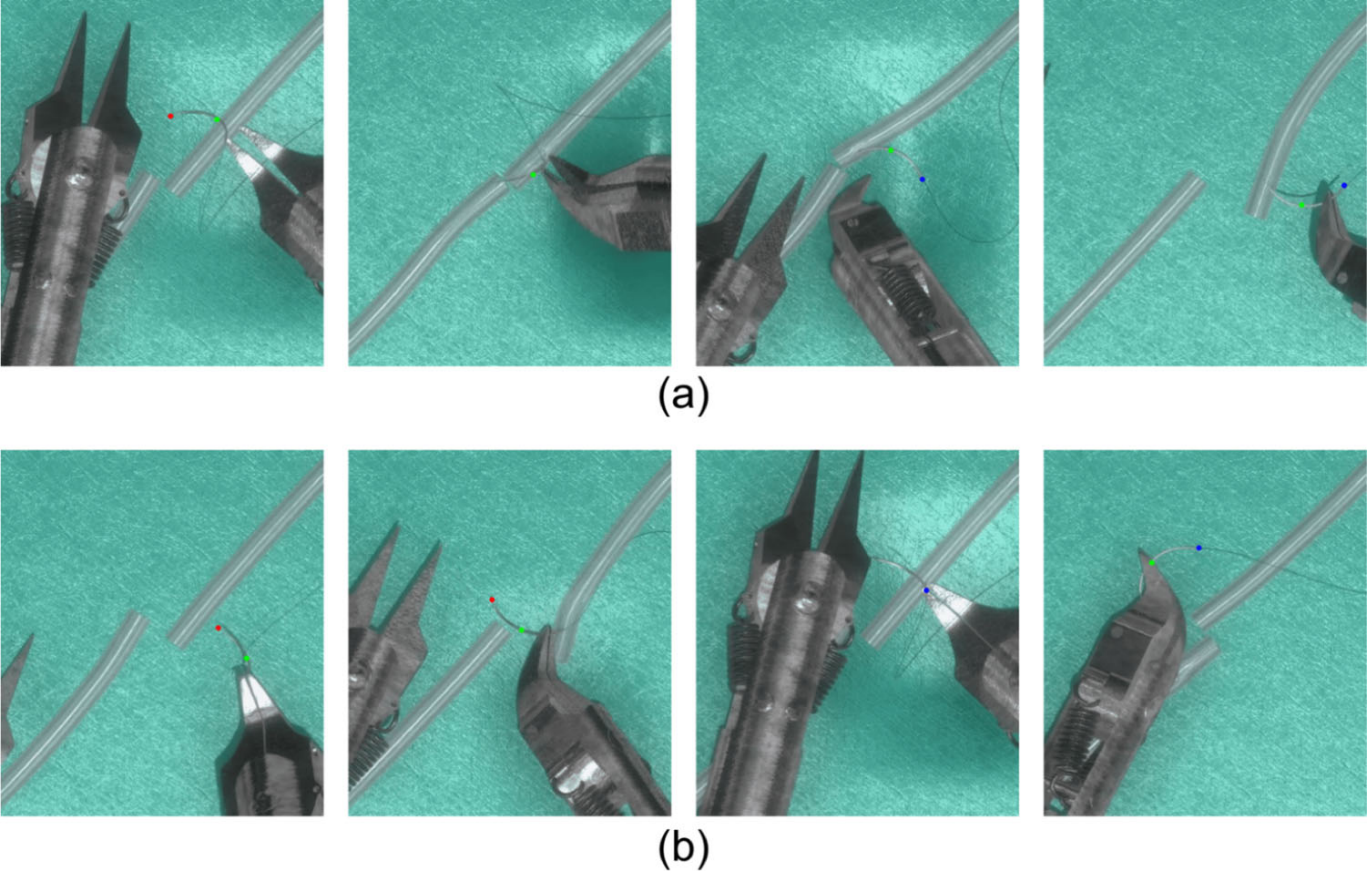
Failure detections: **a** partial occluded by blood vessel during needle insertion, **b** severely occluded by instrument during grasping

For synthetic data, the 2D keypoint translation errors are detailed in Table 1, while Table 2 presents results for 3D orientation. Figure 8 shows the results of 2D keypoints detection and 3D orientation estimation. The average keypoint translation error was within 0.12 mm, and the average angular errors of needle-frame vectors were within $20^{\circ }$. Notably, the model, trained on simulator-generated synthetic data, can predict occluded keypoints (Fig. 9). Missing detections mainly result from (a) partial occlusion by blood vessels during needle insertion and (b) severe occlusion by instruments during grasping (Fig. 10). A video evidencing needle pose estimation on synthetic data is provided as supplementary material.

**Table 2:** 3D orientation error on synthetic data

	Normal vector	Direction vector
Cosine similarity	$0.962 \pm 0.039$	$0.948 \pm 0.056$
Angular error	$12.75^{\circ } \pm 6.19^{\circ }$	$15.55^{\circ } \pm 5.71^{\circ }$

### Results on real data

**Fig. 11:**
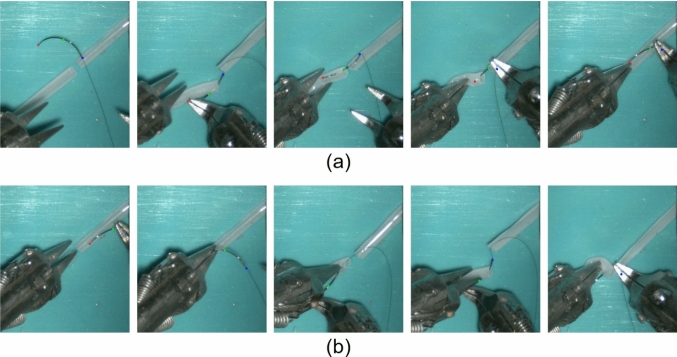
Results of keypoint detection on real images: **a** correct cases, **b** incorrect cases

Quantitative results for 2D keypoint translation errors on real data are presented in Table 3, with visual examples shown in Fig. 11. The average translation error of tip, middle and end point is 1.56, 1.71 and 1.62 pixels, since the ratio $r=\frac{pixel }{real } \approx 33.33$, the translation error in real coordinates is approximately 0.047, 0.051 and 0.049 mm. Keypoints ratio is defined as the proportion of detected keypoints whose translation errors are less than 4 pixels relative to the ground truth, expressed as a percentage. The results indicate that most detection errors arose from occlusion, particularly in cases where manual labeling of ground-truth keypoints was infeasible due to invisibility. A video showing keypoint detection on real images is also included as supplementary material.

**Table 3:** Evaluation results on real images

	Tip point	Middle point	End point
Translation error (pixel)	$1.56 \pm 1.32$	$1.71 \pm 1.86$	$1.62 \pm 1.34$
Equivalent translational error (mm)	$0.047 \pm 0.040$	$0.051 \pm 0.056$	$0.049 \pm 0.041$
Detection ratio	93.87%	89.21%	92.57%
Keypoints ratio (error < 4 px)	96.30%	92.68%	94.79%

### Results of correlation analysis

Pearson correlation analysis results are presented in Table 4. The results revealed strong positive correlations between translation errors and orientation angular errors, with correlation coefficients of 0.79 for the direction vector and 0.72 for the normal vector ($p<0.05$). This highlights the significant impact of keypoint detection accuracy on orientation estimation, emphasizing the need for precise keypoint localization to ensure reliable 3D orientation results.

Furthermore, this correlation analysis provides a practical framework for estimating orientation error in real data, where ground-truth 3D pose information is not directly available. Based on the established relationship from synthetic data, as showed in Table 5, keypoint translation errors below 4 pixels correspond to average angular errors of $8.9^{\circ }$ for the direction vector and $11.2^{\circ }$ for the normal vector. On the real dataset, the average keypoint translation error was under 2 pixels, with 93.85% of keypoints having errors below 4 pixels, indicating orientation errors were within acceptable limits despite occasional inaccuracies caused by occlusion.

**Table 4:** Correlation analysis between keypoints translation error and angular error of frames vectors

Metric	Correlation coefficient (*r*)	*p* value
Translation error vs. angular error (direction vector)	0.79	$<0.05$
Translation error vs. angular error (normal vector)	0.72	$<0.05$

**Table 5:** Relationship of keypoints translation error (pixel) and angular error of frames vectors

Keypoints error range	$e_p \in [0, 2)$	$e_p \in [2, 4)$	$e_p \in [4, 6)$	$e_p \in [6, 8)$	$e_p \in [8, 10)$
Average angular error of $\textbf{d}$	$4.7^{\circ }$	$8.9^{\circ }$	$9.8^{\circ }$	$15.1^{\circ }$	$17.6^{\circ }$
Average angular error of $\textbf{n}$	$6.9^{\circ }$	$11.2^{\circ }$	$13.9^{\circ }$	$17.3^{\circ }$	$20.4^{\circ }$

## Discussion

This study demonstrates the feasibility of an innovative method for estimating the 2D position and 3D orientation of surgical needles from a monocular image, experimental results on synthetic data show promising accuracy, with average keypoint translation error below 0.2 mm. While this corresponds to approximately 29% of a typical small blood vessel diameter (e.g., 0.7 mm), it is important to note that this average includes frames where the needle tip is occluded. During the critical insertion phase. When the needle tip is clearly visible,the error consistently falls below 0.1 mm, which supports precise and reliable needle positioning. Moreover, the estimated needle-frame vectors consistently remained within clinically acceptable angular error thresholds ($\pm 20^{\circ }$), as referenced in prior surgical assessment literatures [28, 29]. These results validate the precision and reliability of the proposed method for robotic surgical applications.

The results on the real data reveal a lower average error (below 0.1 mm) but a reduced detection ratio-$-$93.87%, 89.21%, and 92.57% for the tip, middle, and end points– compared to the detection ratio of synthetic data, which are 94.83%, 98.49%, and 97.38% for the corresponding keypoints. This is primarily because keypoints that were severely occluded in real images (4.69%, 3.47%, 3.99% for the tip, middle and end points) could not be manually annotated, while all keypoints were annotated for the synthetic data. The comparatively higher error observed in the synthetic data is mainly due to the heavily occluded keypoints that were not detected for the real data.

By utilizing high-accurate, annotation-free synthetic data generated through a simulation environment, a convolutional neural network can be trained to generalize effectively to real surgical images, enabling vision-based needle localization without stereo-vision or additional sensors. This represents a significant advancement in addressing a core challenge in robotic microsurgery–accurate tool localization in constrained and visually limited environments. Performance of our method indicates the potential to enhance autonomy in robotic microsurgical anastomosis, particularly by supporting fine-grained, visually guided needle adjustments.

However, several limitations constrain the generalizability and robustness of our method. The approach assumes a fixed needle geometry and static camera calibration–conditions that may not hold in all surgical setups. In addition, performance deteriorates under challenging imaging conditions such as severe occlusions, complex lighting, or strong image noise. To advance this research for full-autonomous robotic microsurgical anastomosis, future work should focus on (1) improving model accuracy by reducing domain discrepancy between synthetic and real images; (2) integrating monocular depth estimation to enable more robust and reliable 6D pose estimation from single-image inputs; and (3) leveraging temporal consistency from video streams to enhance orientation estimates with motion cues.

## Conclusion

This study presents a monocular image-based approach for estimating the pose (2D position and 3D orientation) of a suturing needle using convolutional neural network. By leveraging a hybrid dataset augmented with synthetic images, a CNN was trained to accurately predict keypoints of the needle. The needle’s orientation was estimated from these keypoints and its geometric properties. Our approach addresses the challenges of needle pose estimation in robotic microsurgery, where precise manipulation and tracking are crucial.

Future work will expand validation with larger datasets, improve occlusion handling, and refine performance on real-world data for clinical use. Integrating monocular depth estimation techniques [30] will also be explored for 6D pose estimation, advancing autonomous robotic suturing and surgical skill assessment.

## Supplementary Information

Below is the link to the electronic supplementary material.

Supplementary file 1 (mp4 100489 KB)

Supplementary file 2 (pdf 213 KB)
