# Supplementary material for: Monocular suture needle pose detection using synthetic data augmented convolutional neural network
Source: Int J Comput Assist Radiol Surg. 2025 Jun 24;20(10):2019–30. doi: 10.1007/s11548-025-03467-1 (PMC12518471; doi:10.1007/s11548-025-03467-1)
Supplement: Supplementary file 2 — (pdf 213 KB) [file 11548_2025_3467_MOESM2_ESM.pdf]

Supplementary Materials - Monocular Suture Needle Pose Detection using Synthetic Data Augmented Convolutional Neural Network

Yifan Wang[1*], Saul Alexis Heredia Perez[2] and Kanako Harada[1,2]

*Corresponding author(s). E-mail(s):
wang-yifan971125@g.ecc.u-tokyo.ac.jp;
Contributing authors: saulheredia@g.ecc.u-tokyo.ac.jp;
kanakoharada@g.ecc.u-tokyo.ac.jp;

1 Appendix A: Model Architecture and Implementation Details

A U-Net-inspired encoder-decoder architecture [1] is employed to predict keypoint confidence maps. The input image is first passed through a hierarchically structured encoder to extract multi-scale feature maps. In our implementation, the encoder is based on ResNet-34 [2].

The decoder generates a keypoint confidence map whose dimension is identical to the input image, with the number of channels equal to the keypoints classes. To leverage information from diverse scale features, deconvolution and upsampling are applied to the outputs of Conv5-Conv8, which are then concatenated with Conv4-Conv1, respectively. Finally, the Conv9 output is processed through a convolutional and upsampling layer, producing a confidence map with three channels for the tip, middle, and end points.

Keypoint positions are then obtained by identifying the local maxima in the respective confidence maps, which represent the most probable keypoint locations.

2 Appendix B: Microscopic camera mathematical model for error analysis

To illustrate why the projection lines AA', BB', CC' can be considered as perpendicular to the plane $A'B'C'$, we use the following mathematical model of microscopic camera:

Microscope Specifications: Following are the Zeiss OPMI-MD surgical microscope specifications: focal length $f = 175$ mm, magnification ratio $m = 0.25$. According to [3], the working distance $d_w = f(1 + \frac{1}{m}) = 175 \times (1 + 4) = 875$ mm.

Perspective Projection Model: We define a 3D point $P(x, y, z)$ relative to the microscope's perspective center. Its projection onto the camera sensor corresponds to the pixel coordinates $p(x', y')$. Based on pinhole model, the projection on the camera sensor can be described as:

$$\begin{pmatrix} x' \\ y' \end{pmatrix} = -\frac{f}{z} \begin{pmatrix} x \\ y \end{pmatrix}. \tag{1}$$

Field of View and Depth of Field: For microscopy, the Field of View (FoV) $(\Delta x_{\max}, \Delta y_{\max})$ and the Depth of Field (DoF) $\Delta z_{\max}$ are significantly smaller than the working distance d_w. In this configuration, we have: $d_w = 875$ mm, $|\Delta z_{\max}| = 10$ mm, $|\Delta x_{\max}| = |\Delta y_{\max}| = 40$ mm.

Depth Variation and Pixel Shift: We consider two points with the same x, y coordinates at the extreme depths of the field:

$$P_1 : (x, y, d_w - |\Delta z_{\max}|), P_2 : (x, y, d_w + |\Delta z_{\max}|) \tag{2}$$

Their projections on the sensor are calculated as follows:

$$p_1 = -\frac{f}{d_w - 0.5\Delta z_{\max}}(x, y), \quad p_2 = -\frac{f}{d_w + 0.5\Delta z_{\max}}(x, y) \tag{3}$$

The distance between these two pixel locations is approximately:

$$\left| \frac{175}{870} - \frac{175}{880} \right| (x, y) \approx 0.002(x, y) \tag{4}$$

This displacement is so minimal that both projections are essentially indistinguishable, often falling within the same pixel on the sensor. Thus, it is reasonable to assume that the keypoints and their projections are approximately perpendicular to the image plane.

References

[1] Ronneberger, O., Fischer, P., Brox, T.: U-net: Convolutional networks for biomedical image segmentation. In: Medical Image Computing and Computer-Assisted Intervention–MICCAI 2015: 18th International Conference, Munich, Germany, October 5-9, 2015, Proceedings, Part III 18, pp. 234–241 (2015). Springer

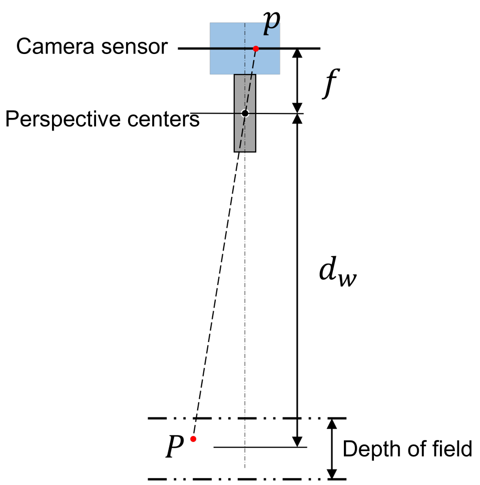

Fig. 1 Illustration of microscopic camera model

[2] He, K., Zhang, X., Ren, S., Sun, J.: Deep residual learning for image recognition. In: Proceedings of the IEEE Conference on Computer Vision and Pattern Recognition, pp. 770–778 (2016)

[3] Lin, X., Heredia Pérez, S.A., Harada, K.: A cranial-feature-based registration scheme for robotic micromanipulation using a microscopic stereo camera system. Advanced Robotics **38**(24), 1730–1742 (2024)
